# Skin immunization by microneedle patch overcomes statin-induced suppression of immune responses to influenza vaccine

**DOI:** 10.1038/s41598-017-18140-0

**Published:** 2017-12-19

**Authors:** Elena V. Vassilieva, Shelly Wang, Song Li, Mark R. Prausnitz, Richard W. Compans

**Affiliations:** 1 Department of Microbiology & Immunology and Emory Vaccine Center, Emory University School of Medicine, Atlanta, GA Georgia; 2 School of Chemical and Biomolecular Engineering, Georgia Institute of Technology, Atlanta, GA Georgia

## Abstract

Recent studies indicated that in elderly individuals, statin therapy is associated with a reduced response to influenza vaccination. The present study was designed to determine effects on the immune response to influenza vaccination induced by statin administration in a mouse model, and investigate potential approaches to improve the outcome of vaccination on the background of statin therapy. We fed middle aged BALB/c mice a high fat “western” diet (WD) alone or supplemented with atorvastatin (AT) for 14 weeks, and control mice were fed with the regular rodent diet. Mice were immunized with a single dose of subunit A/Brisbane/59/07 (H1N1) vaccine, either systemically or with dissolving microneedle patches (MNPs). We observed that a greater age-dependent decline in the hemagglutinin inhibition titers occurred in systemically-immunized mice than in MNP- immunized mice. AT dampened the antibody response in the animals vaccinated by either route of vaccine delivery. However, the MNP-vaccinated AT-treated animals had ~20 times higher total antibody levels to the influenza vaccine than the systemically vaccinated group one month postvaccination. We propose that microneedle vaccination against influenza provides an approach to ameliorate the immunosuppressive effect of statin therapy observed with systemic immunization.

## Introduction

Almost 20% of the US population over 40 years old and nearly half of the population over 75 years old receive statin therapy as an approach to reduce cardiovascular disease through reduction of blood cholesterol^1,2^. Statins decrease synthesis of cholesterol in the liver by inhibiting 3-hydroxy-3-methylglutaryl coenzyme A reductase. Because cholesterol is involved in numerous metabolic pathways, the overall effects of statins are pleiotropic. The effect of statin therapy on influenza outcome in the elderly population has been debated previously^3,4^. Recent studies indicated that in elderly individuals, statin therapy is associated with a reduced response to influenza vaccination^5–7^. This association was based on the reduction of the hemagglutination-inhibiting geometric mean titers (HAI GMT)^5^, increased incidence of medically attended acute respiratory illness^6^ and a higher frequency of laboratory-confirmed influenza^7^ in the vaccinated statin users when compared with non-users. This information is concerning because the aged population, which is a target group for statin therapy, is already at high risk for morbidity and mortality caused by influenza due to immunosenescence^8–11^. Thus, finding a way to overcome statin-induced suppression of immune responses to vaccination in older individuals is an important goal that we have investigated by comparing an alternative route of influenza vaccine delivery to standard systemic vaccination.

Cutaneous antigen delivery^12^ using variety of devices and vaccines^13^ including influenza vaccines^14–20^ is an active and promising area of research with important implications for public health. We^17–19,21–23^ and other investigators^24–28^ have observed improved immune responses to influenza vaccination by MNPs. Improved response to skin-delivered antigens occurs due to a network of immunoregulatory cells in skin^29–31^ including specialized sets of resident antigen-presenting cells (APC)^32^. Activated APCs migrate to the proximal draining lymph nodes where they present vaccine peptides to helper and cytotoxic T cells and interact with B-cells, thus initiating an effective immune response. The uptake of vaccine antigen by a highly motile CD207 (langerin) (+) DC subpopulation of skin APCs was visualized by two-photon microscopy^33^. We have demonstrated that depletion of CD207 (+) dermal DCs prior to vaccination resulted in partial impairment of both Th1 and Th2 responses in microneedle-immunized but not systemically-vaccinated mice^34^ confirming the important role of this subset of APCs in skin vaccination. MNP insertion alone caused local release of proinflammatory cytokines and chemokines, further increased in the presence of influenza antigen. This local innate response induced activation, maturation and migration of antigen – loaded APCs^35^. “Mechanical adjuvant” properties of MNPs^36,37^ are thought to be due to a limited amount of cell death-induced transient local inflammation responsible for increased production of influenza vaccine-specific antibody that correlated with the increased level of cell death^38^.

Our previous studies^23,39–42^ led to a successful Phase I clinical trial of the safety, immunogenicity, reactogenicity and acceptability of the trivalent influenza vaccine delivered with a MNP^43^. We hypothesized that skin-delivery of influenza vaccines will ameliorate the immunosuppressive effect of statin therapy seen with systemic immunization. To test this hypothesis, we compared the outcomes of two routes of immunization in combination with statin treatment: the systemic route by intramuscular injection most widely used in vaccination, and a skin immunization route using MNP. To better model human studies, we used middle-aged mice, administered AT orally on a background of a high-fat WD for 14 weeks prior to immunization, and assayed total cholesterol in blood to confirm that AT treatment affected cholesterol levels prior to vaccination.

## Results

### Age dependency of HAI titers elicited by systemic and MNP vaccine delivery

**Figure 1:**
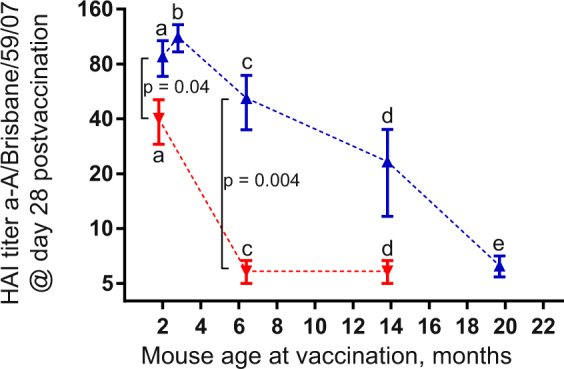
Age-dependent decline of anti-A/Brisbane/59/07 (H1N1) HAI titers measured at day 28 postvaccination with 2.4–3.2 µg vaccine in systemically immunized (red symbols) and skin-immunized (blue symbols) BALB/c mice presented as means with SEM on the log 2 scale. The data from this study are compiled together with previously reported titers and plotted against mouse age at time of immunization. Mouse groups: (a) mice on the RD immunized with MNPs (n = 5, ~2.3 µg HA) or IM (n = 5, 3 µg HA) replotted from^23^, (b) and (e) mice on RD immunized with MNP Batch 2 (n = 8 each time point, 3.2 µg HA), (c) Mice on RD immunized with MNP Batch 1 (n = 6, 2.7 µg HA) or IM (n = 5, 2.4 µg HA), (d) Mice on WD immunized with MNP Batch 1 (n = 6, 2.7 µg HA) or IM (n = 5, 2.4 µg HA). P values calculated by 2 tailed unpaired t-test on the log_2_-transformed titers are shown for the groups in which they were below 0.05. Individual antibody responses for each mouse are shown in Supplemental Fig. 3.

We vaccinated adult (2-3-month-old), mature (6.5-month-old), middle-aged (14-month-old) and advanced aged (20-month-old) mice, none of which received AT, with a single dose of A/Brisbane/59/07 (H1N1) vaccine by IM or MPN delivery and plotted HAI titers detected at day 28 postvaccination against mouse age (Fig. 1). The highest titers around HAI 80 were observed in the adult mice vaccinated with MNPs, while in the IM-vaccinated animals of the same age they were 2-fold lower (p = 0.04). The titers in both groups declined with age, but the decline was more pronounced in the systemically vaccinated mice. MNP groups demonstrated significantly higher titers than IM groups until at least 6.5 month of age at the time of vaccination (p = 0.004). In mice vaccinated at 14 months, HAI titers above the detection limit of 10 were observed in ~70% of animals in MNP groups, but only in ~20% in the IM groups (Fig. 1). Thus, MNP vaccination decreased the age – dependent decline of the functional antibody titers observed in the systemically immunized mice.

### AT decreases total blood cholesterol

**Figure 2:**
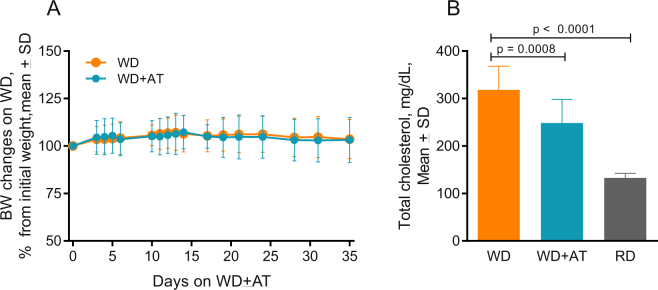
Effects of WD and AT on mouse weight and blood cholesterol. (**A**) Effect on body weight. Mice were fed RD until they were 10.5 months old and then switched to the high fat WD with or without AT. The individual weights are normalized to the weight at the time of the diet switch (time zero on the graph) and presented as means with SD (n = 18 for WD, n = 17 for WD + AT); (**B**) Effect of WD and AT consumed for 7 weeks on the total cholesterol level measured in mouse blood. Values are expressed as means with SD (n = 18 for WD, n = 13 for WD + AT, n = 5 for RD). Data points for individual mice are shown in Supplemental Fig. 4.

Within two weeks after starting the WD alone or with AT, the mice gained an average of 6-7% of their original body weight (p < 0.05), and by the fifth week the weight stabilized at 103% of the initial weight, although the increase was not statistically significant (Fig. 2A). Independent of AT treatment, all mice on the high fat WD displayed oily fur compared to the mice on the RD. Thus, AT did not affect the weight or the fur appearance of mice on the WD. We observed that consumption of the WD for 7 weeks elevated the level of total cholesterol in blood by 140% compared to the mice kept on the RD (p < 0.0001, Fig. 2B). AT incorporated into the WD lowered total plasma cholesterol by 22% (p = 0.0006). Thus, the mouse model reproduced the main effects of a high fat diet and statin treatment on cholesterol levels observed in humans.

### AT dampens antibody responses in immunized mice

**Figure 3:**
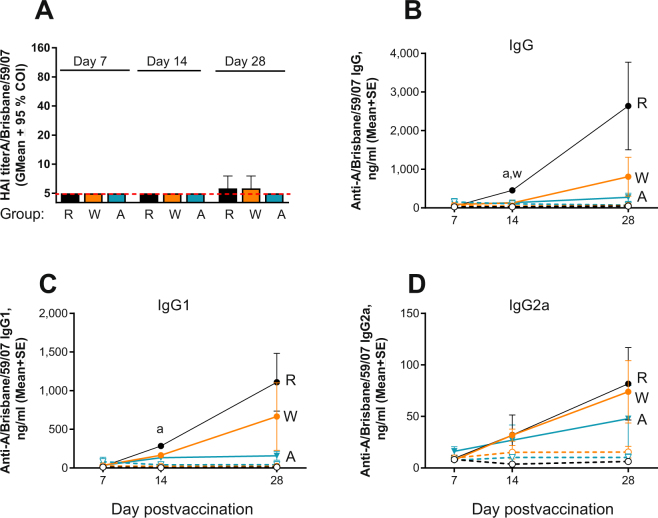
Effect of AT on the antibody response to vaccination in systemically immunized groups. Groups: R - RD (n = 6, black symbols), W - WD (n = 6, orange symbols), A - AT (n = 5, blue symbols): (**A**) HAI titers measured at days 7, 14 and 28 post immunization. Samples below limit of detection including all naïve samples (n = 4 in RD, n = 6 in WD, n = 5 in AD) were assigned a titer of 5 (red broken line) for calculations. Data are presented as geometric means with 95% confidence intervals. (**B**–**D**) Total vaccine-specific IgG, IgG1, and IgG2a, respectively. Filled and open circles connected with solid or broken lines represent immunized and naïve groups, respectively. Data are presented as means with SE where “a” and “w” denote statistically significant differences between the RD group and AT or WD groups, respectively: p = 0004 and 0.001 for “a” and “w”, respectively, at d 14 on panel B; p = 0.016 and 0.049 for “a” at day 14 and 28, respectively on panel C. Detailed antibody responses of individual mice are shown in Supplemental Fig. 5.

The HAI titers elicited by the vaccine were below the detection level of 10 in all naïve and systemically immunized mice except for one mouse with HAI = 10 detected in both WD and RD groups at d 28 postvaccination (Fig. 3A). This is not an unexpected result given the single vaccination dose and age of the animals (groups “d” in Fig. 1). The vaccine - specific IgG level in blood was found to be ~3.5 times higher in the mature RD group (6.5-month-old mice at the time of vaccination, groups “c” in Fig. 1) than in either the WD or AT groups (p = 0.01 and 0.004, respectively) at 2 weeks postvaccination (Fig. 3B). Similarly, IgG1 levels were two times (p = 0.016) and seven times (p = 0.049) higher in RD group than in the AT group at 2 and 4 weeks postvaccination, respectively (Fig. 3C). IgG, IgG1, and IgG2a levels in the WD group were up to four times higher than in the AT group (Fig. 3B and C), but there was no statistically significant difference between the groups. These results indicate a trend of reduction in vaccine-specific IgG, IgG1, and IgG2a levels in the middle aged systemically vaccinated mice receiving AT, and low overall antibody titers.

**Figure 4:**
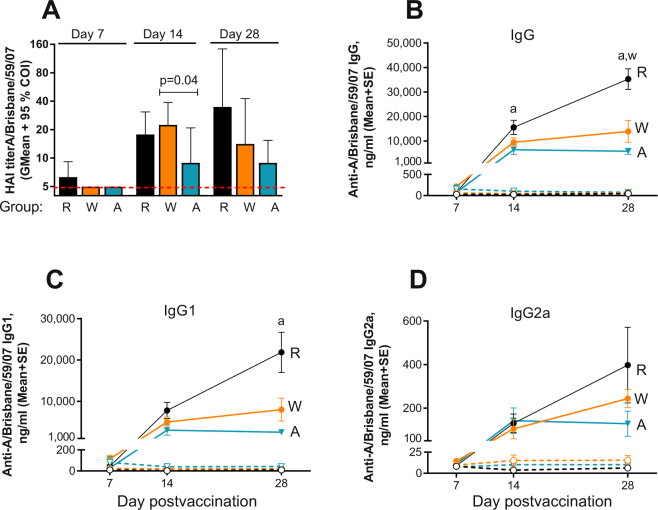
Effect of AT on the antibody response to vaccination in MNP groups. (**A**) HAI presented as described in Fig. 3. Groups: R - RD (n = 6, black symbols), W - WD (n = 6, orange symbols), A - AT (n = 6, blue symbols). (**B**–**D**) Total vaccine-specific IgG, IgG1, and IgG2a, respectively. Filled and open circles connected with solid or broken lines represent immunized and naïve groups, respectively. “a” and “w” represent differences described in the legend for Fig. 3: p = 0.026 for “a” at day 14, p < 0.001 and p = 0.006 for “a” and “w” respectively at 28 on the panel B; p = 0.0026 for “a” on panel C. Detailed antibody responses of individual mice are shown in Supplemental Fig. 6.

HAI titers of 10 or above were detected in all MNP-vaccinated RD and WD groups and in 50% of the AT group as soon as 2 weeks post immunization (Fig. 4A). The mature mice on the RD developed the highest titers among other groups (GMT 34.8 at 4 weeks postvaccination, Fig. 4A), while the highest HAI titers for the middle aged mice on WD (GMT 22.4) were observed in the group that did not receive AT on day 14 postvaccination. Addition of AT to the WD decreased this number by 2.5 fold to GMT 8.9 (p = 0.04). The middle aged mice in both MNP groups demonstrated a slight drop in the HAI titers from week 2 to week 4 postvaccination in contrast to the mice on the RD (Fig. 4A). IgG levels in the RD group were 2.5 fold higher (p = 0.006) than in the WD group at day 28 postvaccination and 2.4 (p = 0.026) and 6 fold (p < 0.001) higher than in the AT group at days 14 and 28 postvaccination, respectively (Fig. 4A), while IgG1 was about 9 fold higher than in the AT group at day 28 postvaccination (p = 0.0026, Fig. 4C). AT decreased total vaccine-specific IgG, IgG1 and IgG2a in comparison with the WD MNP groups by 2.4, 2.7, and 2 -fold by day 28 respectively, although the differences were not statistically significant (Fig. 4B–D).

### MNP vaccine delivery enhances the humoral immune response in AT-treated mice

**Figure 5:**
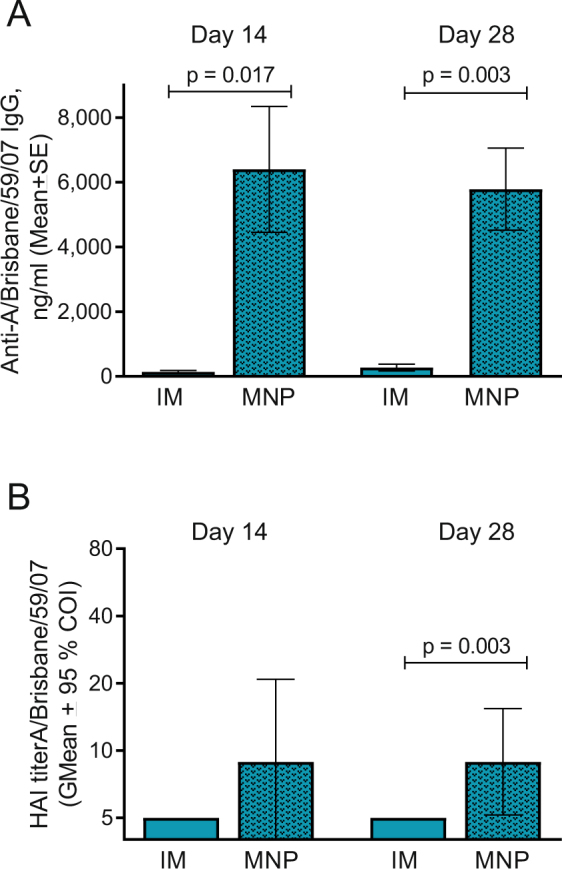
MNP vaccination of AT-treated animals elicited higher antibody response than IM vaccination. (**A**) Comparison of the total vaccine-specific IgG at days 14 and 28 postvaccination in the IM (blue bar, replotted from Fig. 3) and MNP (patterned blue bar, replotted from Fig. 4) groups. (**B**) Comparison of HAI titers measured at days 14 and 28 postvaccination, same groups as on A. Individual data points for each mouse are shown in Supplemental Fig. 7.

Our hypothesis is that skin immunization of statin–treated mice using MNPs will overcome the attenuation of antibody responses observed after systemic immunization. Thus, we compared side by side the total (IgG) and functional (HAI) vaccine-specific antibody titers for the two groups (AT-MNP vs. AT-IM) at weeks 2 and 4 postvaccination (Fig. 5). Comparison of the total IgG titers (Fig. 5A) demonstrated a clear enhancement due to MNP vaccine delivery: total vaccine-specific IgG was 47 fold (p = 0.017) and 21 fold (p = 0.003) higher in the MNP-vaccinated animals than in the systemically vaccinated animals on the AT regimen by weeks 2 and 4 postvaccination, respectively (Fig. 5A). HAI titers were low because of the animals’ age, but a statistically significant 1.6-fold increase of GMT in the MNP group was observed at day 28 postvaccination (p = 0.037, Fig. 5B). This side-by-side comparison demonstrates that MNP delivery of vaccine overcomes the attenuation of antibody responses caused by AT in the middle aged mice on the WD.

## Discussion

Here, we investigated effects and outcomes of statin therapy in a BALB/c mouse model of influenza immunization. Mouse models of influenza^44^ have been widely used in vaccine research, but not in the context of statin therapy. Mice lack any preexisting immunity or prior exposure to influenza virus, and thus vaccination results in a primary immune response. In contrast, immunization in humans occurs on a background of immunological memory resulting from prior immunizations and infections. Such complexity, together with the co-morbidities often present in the older population, is a reason for some uncertainties noted in human studies^45–47^. The mouse model provides improved control over variables that affect the complexity of the immune response in humans, such as individual variation in infection history and health status. The molecular mechanism of aging in mice is similar to that in humans^48^ and they are considered an ideal model for aging studies based on the concordance of quantitative trait loci identified by genome mapping of mice and humans^49^. Especially important for vaccine research, mice have similar responses to vaccination as humans and age-dependent changes in B and T cells are reproduced in the mouse model^50,51^. Age-related decreased potency of antibody response to vaccination occurs due to deficiencies in generation of plasmablasts induced by vaccination^52^ and defects in T cell responses^53,54^. Studies in aged mice have demonstrated diminished T cell and B cell responses to influenza that closely resembled the responses in elderly humans^55,56^. It is also known that suppressive effects of Treg cells are enhanced with aging^57^, and decreased antigen-specific B cell stimulation in aged mice was associated with elevated levels of a regulatory subset of effector Tregs and defective Tfh cell function^58^.

We started mice on the high fat diet with or without AT more than three months prior to vaccination and observed an AT-dependent decrease in total cholesterol level in blood. AT has previously been shown to decrease total cholesterol in C57BL/6J mice when given with an atherogenic diet, but not with a regular rodent diet^59^, although AT given with the normal rodent diet in approximately 10-fold higher dose than in our study did decrease plasma cholesterol by 26% in 2 weeks^60^. The dose of AT that we used corresponded to that used in previous reports^61^. By using a prolonged statin regimen, high fat diet and middle aged mice, we were able to show that chronic administration of AT considerably decreased vaccine-induced antibody titers. Our mouse data are similar to recent data from human studies which indicate that statins, especially synthetic ones such as AT, reduce antibody responses to influenza vaccination^5–7^.

We found that MNP administration of subunit vaccine increased vaccine-specific antibody titers in the AT-treated mice by ~20 fold compared to IM-immunized mice on the same regimen (p = 0.003) and by ~7 fold (P = 0.0004) compared to the IM-vaccinated mice on a WD diet that did not receive AT. The functional HAI titers that are often used as correlate of protective immunity were as low as 14 (GMT) in the mice on the WD in the MNP-immunized groups and below the level of detection in the IM-groups one month postimmunization, most probably due to the age (14 month) of the animals by the time of immunization. Similarly to a recent report^62^ we demonstrated that the antibody response to vaccination depends on mouse age. Importantly, we found that the age-dependent decline in HAI titers is reduced in skin-immunized mice compared with systemically-immunized mice. AT dampened antibody titers further in both groups. One possible conclusion from this observation is that vaccinees of more advanced age may need an adjuvant in addition to the skin delivery route to boost the humoral response. Co-delivery of an adjuvant and the vaccine formulated in a MNP^63,64^ would be especially attractive because in this case the adjuvant will be administered locally.

A dampening of influenza vaccine-specific antibody titers by AT was previously observed in human studies, and we found a similar trend in the mouse model. The interplay between statin therapy and the outcome of influenza vaccination most likely is a combination of the effect of statins on the host response including cell-mediated and innate immunity, the age of the host and the particular vaccine strain used. Here, we observed that a change from a systemic to a cutaneous route using MNPs has the potential to improve immune responses to existing vaccines which are otherwise compromised by statin therapy.

## Materials and Methods

### Vaccine and microneedle patches

The cell-grown influenza A/Brisbane/59/07 (H1N1) vaccine monobulk was kindly provided by Seqirus (Cambridge, MA). It was concentrated and assayed for protein and hemagglutinin content as previously described^23^. The dissolving MNPs were prepared essentially as described previously^23^ except that polyvinyl alcohol was used as backing material^65^. Specifically, the MNPs were made using polyvinyl alcohol and carobxymethyl cellulose (to provide mechanical strength), sucrose (to stabilize the vaccine and enable rapid dissolution) and potassium phosphate buffer (to control pH). The morphology of MNPs was examined by observation under a microscope immediately after the MNPs were fabricated. Each MNP consisted of 100 microneedles in a 10 × 10 pattern, and each microneedle had a sharp tip measured around 700 µm in length and 200 µm in base diameter. The tips were sharp and straight, indicating sufficient drying of materials (Supplementary Fig. 1S). After immunization the used MNPs were examined again using the same microscopy conditions for the presence of the vaccine-loaded microneedle tips. All microneedle tips disappeared and only the residue bases were left on the patch backing and it was thus concluded that all MNPs resulted in vaccine delivery (Supplementary Fig. 1S). For antigen quantification, the vaccine was extracted from unused and used MNPs for 20 minutes in PBS and the extracts were assayed by ELISA (Supplementary Method). Two batches of MNPs used in this study were loaded with 3.4 and 3.8 µg of HA (hemagglutinin) and 2.7 and 3.2 µg of HA was delivered into the skin, respectively. Thus, the delivery efficiency calculated from the initial and the residual amount of antigen (Supplementary Fig. 2S) was 79.8 ± 8% in the batch 1 and 83.5 ± 6.5% in the batch 2.

### Animals

Female BALB/c mice were obtained from Harlan Laboratories and fed with the regular rodent chow diet (RD) (Laboratory Rodent Diet 5001 (~13% of energy comes from fat, cholesterol ~0.02%), LabDiet, St. Louis, MO) until they were 10.5 month old. Then they were switched for 14 weeks to a high fat rodent WD (Anhydrous Milkfat (20%, cholesterol 0.2%, 1/2” soft pellets; ~40% energy comes from fat), from BioServ (Flemington, NJ) with or without AT. Mice were 14 months old by the time of immunization (middle aged mice). Female BALB/c mice fed with RD were 6.5 months old (mature) by the time of immunization. All animals including naïve animals were kept on the specified diets for the duration of the study. Mice were housed in microisolators with filter tops in a biocontainment level BSL-1 facility and subjected to 12/12 hour light/dark cycle and temperature between 20–22 °C. Three month old (adult) female BALB/c mice from Envigo and 20 month-old (advanced aged) female BALB/cBy mice obtained through the National Institute of Aging were fed with the RD and used in the experiment on age dependency of the immune response. All institutional and national guidelines for the care and use of laboratory animals were followed in accordance with and approved by the Institutional Animal Care and Use Committee (IACUC) at Emory University.

### Administration of atorvastatin

Atorvastatin Ca salt (AK Scientific, Union City, CA), was formulated in the high fat WD by BioServ at 40 mg/kg, which corresponded to 10 mg/kg b.wt per day (~0. 2 mg/day/mouse) assuming an average daily food intake of 5 g per mouse and average mouse weight of 20 g.

### Cholesterol assay

Total cholesterol was measured in blood of fasted mice using a total cholesterol assay kit (Cell Biolabs, San Diego, CA).

### Immunization and sample collection

Mice were immunized once with a dose containing 2.4 µg HA of A/Brisbane/59/07 (H1N1) vaccine systemically by injection of 0.05 ml into the upper quadrant of the hind leg or through skin with batch 1 MNPs that delivered 2.7 µg of vaccine as described^23^. Unvaccinated animals consuming the same diet as the immunized groups were used as controls. Blood was collected at indicated intervals post-immunization and serum was stored at −20 °C until analysis. The mice used in the experiment on age-dependency of the immune response were kept on the RD and vaccinated with 3.2 µg (delivered) of the same vaccine using MNPs from batch 2.

### Humoral immune responses

Vaccine-specific total antibody levels in blood were determined by quantitative ELISA as previously described^23^. Hemagglutination inhibition **(**HAI) titers were assessed based on the WHO protocol^66^ using turkey red blood cells (LAMPIRE, Pipersville, PA). The samples below the lowest level of detection (HAI = 10) were assigned a titer of 5 for calculations. Influenza virus A/Brisbane/59/07 H1N1, generously provided by CDC, was grown in MDCK cells and assayed for hemagglutination (HA) activity as described^66^.

### Statistics

The statistical significance was calculated for selected groups by two-tailed unpaired t-test and *p* ≤ 0.05 was considered significant. HAI titers were converted to log_2_ titers for statistical analysis.

### Data Availability

All data generated or analyzed during this study are included in this published article (and its Supplementary Information files).

## Electronic supplementary material

Supplementary Information
