# Supplementary material for: Skin immunization by microneedle patch overcomes statin-induced suppression of immune responses to influenza vaccine
Source: Sci Rep. 2017 Dec 19;7:17855. doi: 10.1038/s41598-017-18140-0 (PMC5736694; doi:10.1038/s41598-017-18140-0)
Supplement: Supplementary file 1 — Supplementary Information [file 41598_2017_18140_MOESM1_ESM.pdf]

Supplementary information for

Skin immunization by microneedle patch overcomes statin-induced suppression of immune responses to influenza vaccine

Elena V. Vassilieva, Shelly Wang, Song Li, Mark R. Prausnitz, and Richard W. Compans

Supplementary method of antigen quantification in the MNP by ELISA

The content of vaccine was quantified using antigen-captured ELISA as described previously[1] with minor modification. Influenza A/Brisbane/59 specific antibody (H1-Ab-0902A, FDA/CBER, Rockville, MD) was used as both capture and detection antibodies. The antibody was diluted 1:1000 in 0.05M carbonate/bicarbonate buffer (pH9.6), and filled into NUNC MaxiSorp 96-well plates (Thermo Fisher, Rochester, NY), and incubated at 4°C overnight. The antibody-coated plates were washed three times with wash buffer (PBS with 0.05% Tween-20) following by blocking with blocking and the same buffer containing 3% bovine serum albumin at 37°C for 1 hour. After washing the plates another three times, 100 µl of antigen was added into each well and incubated at 37°C for 2 hours. The vaccine solution with known hemagglutinin content measured by single radial immunidiffusion assay[2,3] was used as standard. The horseradish peroxidase (HRP) labelled detection antibody was prepared in advance using the Lightning Link labeling kit (Novus Biologicals). The HRP labelled antibodies were diluted 1:1000 in dilution buffer, and then 100 µl per well added to the washed antigen-captured plates following by incubation at 37°C for 1 hour. The plates were then washed three times, and developed by adding 100 ul per well of SureBlue Reserve TMB Microwell Peroxidase Substrate (1-Component) (KPL, Gaithersburg, MD). After incubation at 37°C for 15 min, the reaction was stopped by adding 100 µl per well of TMB BlueSTOP Solution (KPL), and the absorbance was measured at 620 nm.

Figure 1 Supplement

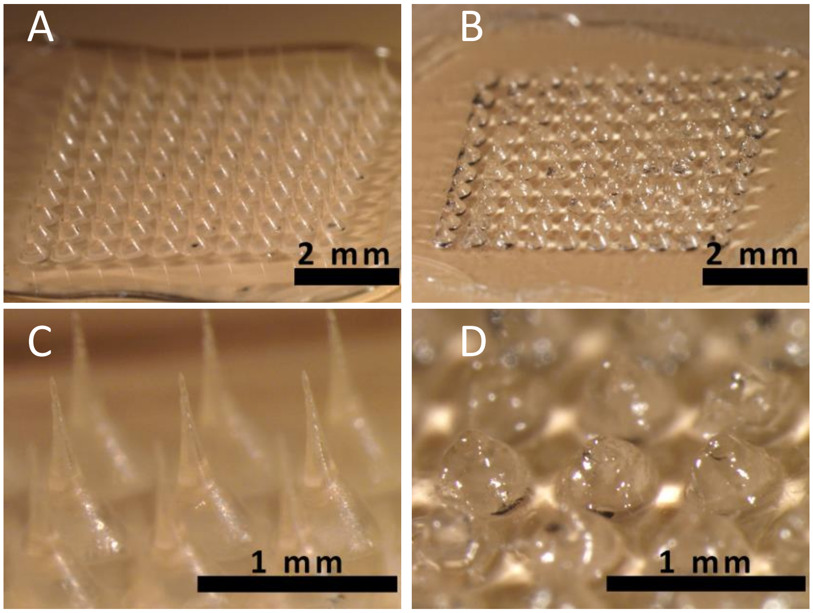

Figure S1, Characterization of MNPs loaded with A/Brisbane/59/07 vaccine. Microphotographs of the MNPs before (A, C) and after (B, D) insertion into the mouse skin. Note dissolution of the sharp microneedle tips in the used MNPs.

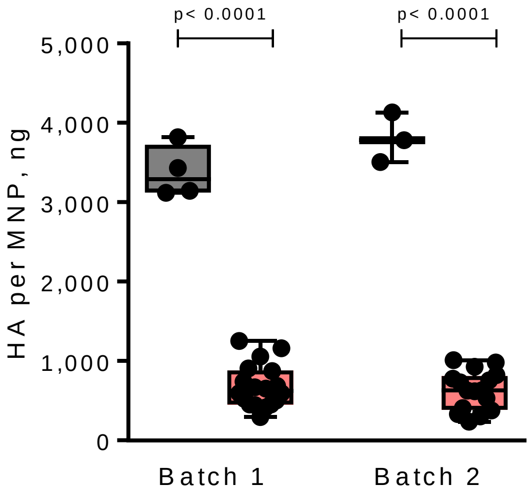

Figure S2. Antigen content of the two batches of MNPs used in the study. The vaccine was extracted from the unused MNPs (black bars, n = 4 batch 1, n = 3 batch 2) and from the used MNPs (pink bars, n = 18 batch 1, n = 16 batch 2) and measured by ELISA (above). The results show single measurements from two independent experiments presented in box and whisker plot with the line indicating the median, box limits representing the upper and lower quartiles and the whiskers showing minimum to the maximum values. The delivery efficiency calculated from these data was 79.8 $\pm$ 8 % in the batch 1 and 83.5 $\pm$ 6.5 % in the batch 2.

Supplementary figures showing individual data points

Figure 3 Supplement

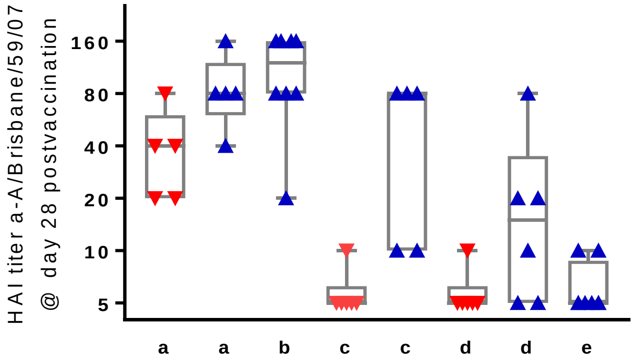

Figure S3. Supplemental data for Fig. 1: individual anti-A/Brisbane/59/07 (H1N1) HAI titers measured at day 28 postvaccination with 2.4-3.2 μg vaccine in systemically immunized (red symbols) and skin-immunized (blue symbols) BALB/c mice presented in box and whisker plot as described for Fig. S2. The letters on the X axis denote the same groups as in Fig.1

Figure 4 Supplement

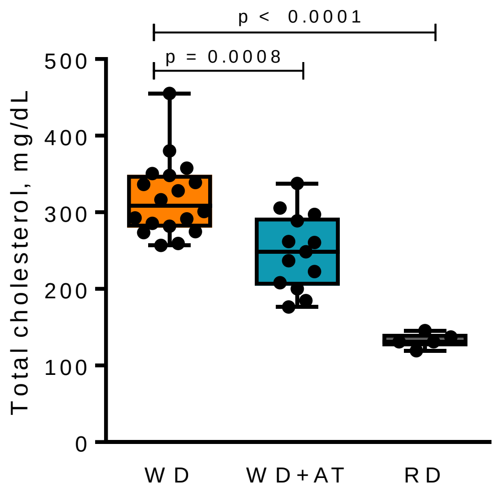

Figure S4. Supplemental data for Fig. 2B: individual measurements of the total cholesterol level in mouse blood presented in box and whisker plot as described for Fig. S2. Results represent a single experiment. The groups are the same as in Fig. 2B.

Figure 5 Supplement

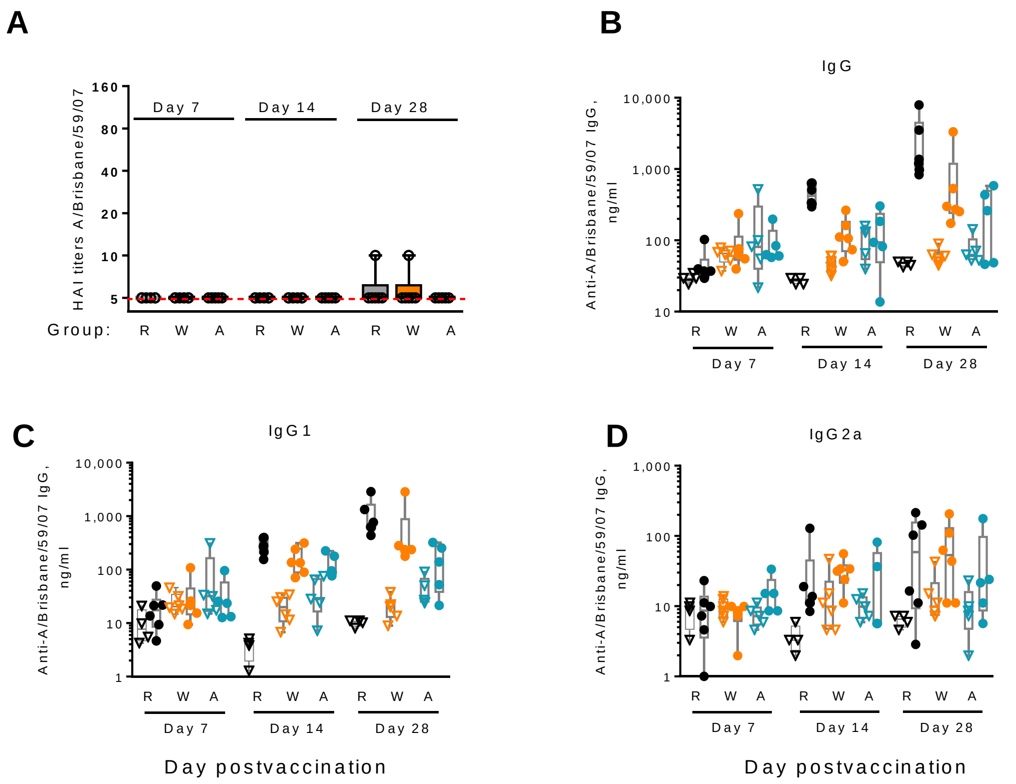

Figure S5. Supplemental data for Fig. 3: Individual HAI titers (A) and total vaccine-specific IgG, IgG1, and IgG2a (B-D) for systemically-vaccinated mice where triangles and dots represent naive and immunized animals, respectively. Data are presented in box and whisker plot as described for Fig. S2. HAI data represent the results of one titration experiment, ELISA data are combined from 2 to 3 measurements for each Ig isotype where each data point represents a single animal. The groups are the same as in Fig. 3.

Figure 6 Supplement

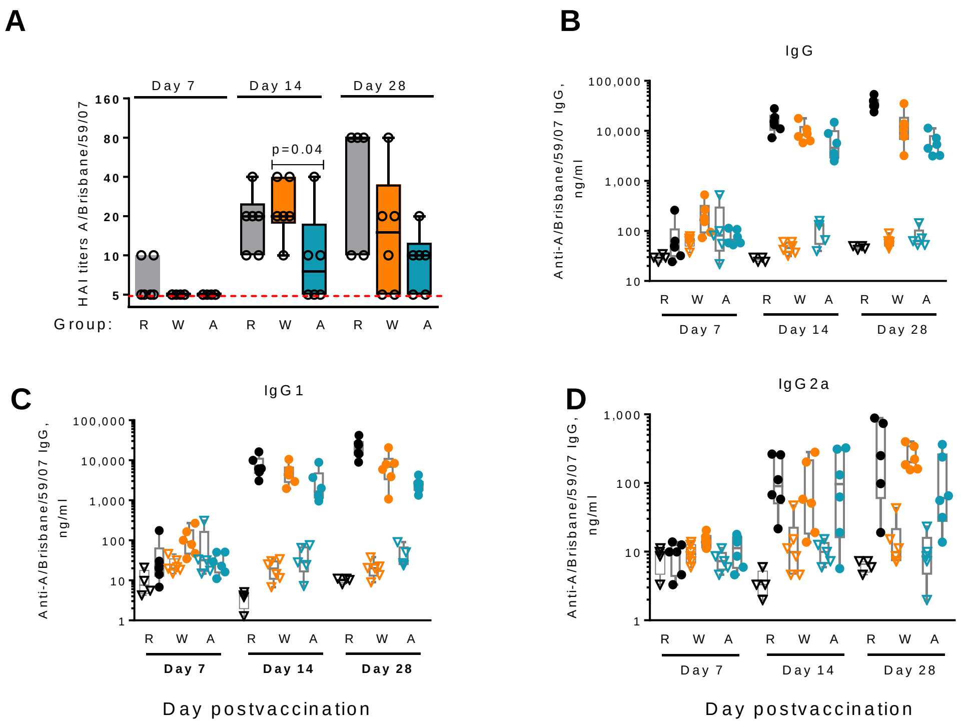

Figure S6. Supplemental data for Fig. 4: Individual HAI titers (A) and total vaccine-specific IgG, IgG1, and IgG2a (B-D) for MNP-vaccinated mice where triangles and dots represent naive and immunized animals, respectively. Data are presented in box and whisker plot as described for Fig. S2. HAI data represent the results of one titration experiment, ELISA data are combined from 2 to 3 measurements for each Ig isotype where each data point represents a single animal. The groups are the same as in Fig. 4.

Figure 7 Supplement

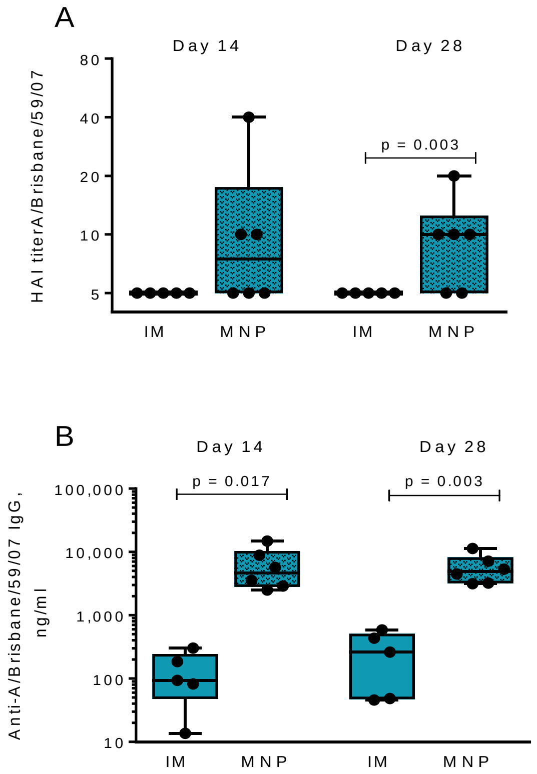

Figure S7. Supplemental data for Fig. 5: individual HAI titers (A) and total vaccine-specific IgG, (B) for IM and MNP-vaccinated AT-treated mice are presented in box and whisker plot as described for Fig. S2. HAI data represent the results of one titration experiment, ELISA data are combined from 2 measurements where each data point represents a single animal. The groups are the same as in Fig. 5.

References

1 Edens, C., Collins, M. L., Goodson, J. L., Rota, P. A. & Prausnitz, M. R. A microneedle patch containing measles vaccine is immunogenic in non-human primates. *Vaccine* **33**, 4712-4718 (2015).

2 Williams, M. S. Single-radial-immunodiffusion as an in vitro potency assay for human inactivated viral vaccines. *Vet. Microbiol.* **37**, 253-262 (1993).

3 Vassilieva, E. V. *et al*. Improved immunogenicity of individual influenza vaccine components delivered with a novel dissolving microneedle patch stable at room temperature. *Drug delivery and translational research* **5**, 360-371 (2015).
